# Supplementary material for: Thermal desorption as a high removal remediation technique for soils contaminated with per- and polyfluoroalkyl substances (PFASs)
Source: PLoS One. 2020 Jun 25;15(6):e0234476. doi: 10.1371/journal.pone.0234476 (PMC7316335; doi:10.1371/journal.pone.0234476)
Supplement: S1 Appendix — (DOCX) [file pone.0234476.s001.docx]

**Supporting Information**

# Thermal desorption as an effective remediation technique for soil contaminated with per- and polyfluoroalkyl substances (PFASs)

# M. Sörengård^a,*^, A-S. Lindh^a^, L. Ahrens^a^

^a^Department of Aquatic Sciences and Assessment, Swedish University of Agricultural Sciences (SLU), Box 7050, SE-750 07 Uppsala, Sweden

**^*^**mattias.sorengard@slu.se

**Table S1. Averarage concentrations [mg PFAS/kg dry weight (dw)] and standard deviations (SD) of experimental triplicates as non-contaminated positive blanks (present in oven during treatments) at different treatment temperatures using clay soil from Vreta kloster, Sweden**

***
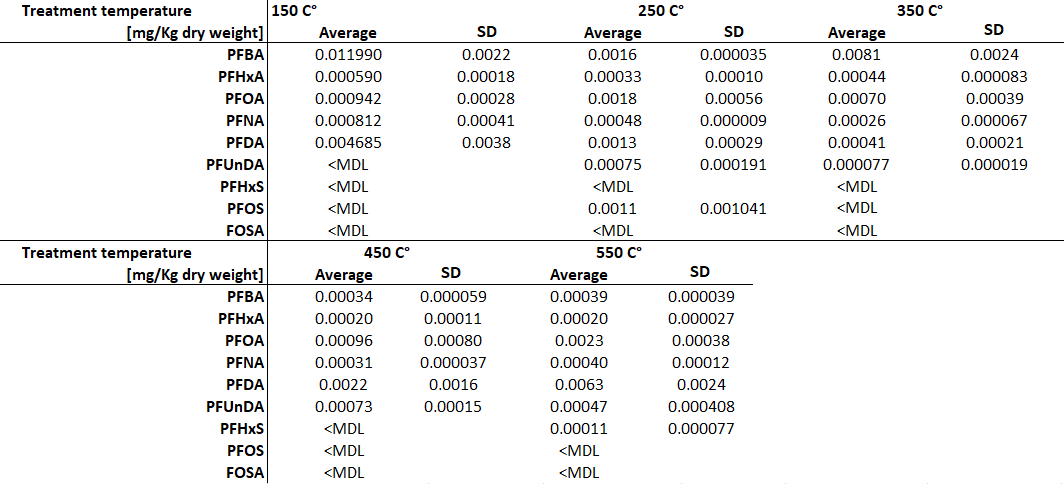
***

**Table S2. Averarage concentrations [mg PFAS/kg dry weight (dw)] and standard deviations (SD) of experimental triplicates after different treatment temperatures and times in clay soil from Vreta kloster, Sweden, fortified with 0.6 mg PFAS/kg dry dw**

| **Treatment** | **Minutes** | **PFBA** | | **PFHxA** | | **PFOA** | | **PFNA** | |
| --- | --- | --- | --- | --- | --- | --- | --- | --- | --- |
| **temperature** | **of treatment** | **[mg/kg dw]** | **SD** | **[mg/kg dw]** | **SD** | **[mg/kg dw]** | **SD** | **[mg/kg dw]** | **SD** |
| **150°C** | **15** | 0.35 | 0.023 | 0.38 | 0.014 | 0.89 | 0.087 | 0.54 | 0.031 |
|  | **45** | 0.510 | 0.059 | 0.38 | 0.031 | 0.86 | 0.070 | 0.51 | 0.055 |
|  | **75** | 0.4109 | 0.031 | 0.43 | 0.034 | 0.72 | 0.064 | 0.52 | 0.029 |
| **250°C** | **15** | 0.47 | 0.096 | 0.49 | 0.101 | 0.73 | 0.068 | 0.56 | 0.11 |
|  | **45** | 0.14 | 0.021 | 0.17 | 0.011 | 0.30 | 0.025 | 0.28 | 0.037 |
|  | **75** | 0.052 | 0.013 | 0.070 | 0.010 | 0.14 | 0.011 | 0.13 | 0.021 |
| **350°C** | **15** | 0.059 | 0.00080 | 0.043 | 0.0030 | 0.076 | 0.0030 | 0.060 | 0.0032 |
|  | **45** | 0.010 | 0.0006 | 0.019 | 0.0034 | 0.026 | 0.0070 | 0.025 | 0.0051 |
|  | **75** | 0.0075 | 0.0016 | 0.0046 | 0.00060 | 0.0048 | 0.00030 | 0.0029 | 0.00050 |
| **450°C** | **15** | 0.011 | 0.0026 | 0.013 | 0.0012 | 0.017 | 0.0030 | 0.018 | 0.00030 |
|  | **45** | 0.0040 | 0.0016 | 0.0011 | 0.00090 | 0.00050 | 0.00010 | 0.00040 | 0.00020 |
|  | **75** | 0.0087 | 0.0080 | 0.0011 | 0.00090 | 0.00040 | 0.00010 | 0.00050 | 0.00010 |
| **550°C** | **15** | 0.0040 | 0.0010 | 0.00050 | 0.00010 | 0.00030 | <MDL | 0.00040 | 0.00010 |
|  | **45** | 0.0016 | 0.00030 | 0.00030 | 0.00010 | 0.00050 | 0.00010 | 0.00040 | 0.00010 |
|  | **75** | 0.0018 | 0.001 | 0.00040 | 0.00020 | 0.0013 | 0.00040 | 0.0010 | 0.00020 |
|  |  | **PFUnDA** |  | **PFHxS** |  | **PFOS** | | **FOSA** | |
|  |  | **[mg/kg dw]** | **SD** | **[mg/kg dw]** | **SD** | **[mg/kg dw]** | **SD** | **[mg/kg dw]** | **SD** |
| **150°C** | **15** | 0.23 | 0.040 | 0.472+ | 0.041 | 1.0 | 0.28 | 1.4 | 0.18 |
|  | **45** | 0.31 | 0.066 | 0.56 | 0.072 | 0.88 | 0.069 | 0.85 | 0.021 |
|  | **75** | 0.54 | 0.16 | 0.50 | 0.020 | 1.1 | 0.059 | 0.78 | 0.084 |
| **250°C** | **15** | 0.54 | 0.092 | 0.50 | 0.022 | 0.99 | 0.13 | 1.3 | 0.95 |
|  | **45** | 0.54 | 0.064 | 0.52 | 0.022 | 0.86 | 0.060 | 0.48 | 0.038 |
|  | **75** | 0.20 | 0.055 | 0.49 | 0.028 | 1.0 | 0.12 | 0.29 | 0.0088 |
| **350°C** | **15** | 0.15 | 0.063 | 0.47 | 0.058 | 1.1 | 0.046 | 0.34 | 0.025 |
|  | **45** | 0.020 | 0.0053 | 0.52 | 0.030 | 0.78 | 0.035 | 0.018 | 0.0044 |
|  | **75** | 0.0035 | 0.00060 | 0.42 | 0.013 | 0.86 | 0.12 | 0.0040 | 0.00050 |
| **450°C** | **15** | 0.032 | 0.0065 | 0.49 | 0.0080 | 0.73 | 0.030 | 0.052 | 0.026 |
|  | **45** | 0.00060 | 0.00080 | 0.0013 | 0.00030 | 0.0021 | 0.00090 | <MDL |  |
|  | **75** | 0.0013 | 0.00090 | <MDL |  | <MDL |  | <MDL |  |
| **550°C** | **15** | <MDL | <MDL | 0.00020 | 0.00010 | 0.00020 | 0.00020 | <MDL |  |
|  | **45** | 0.00015 | 0.00020 | <MDL |  | <MDL |  | <MDL |  |
|  | **75** | <MDL | <MDL | <MDL |  | <MDL |  | <MDL |  |

**Table S3. Averarage concentrations [mg PFAS/kg dry weight (dw)] and standard deviations (SD) of experimental triplicates after different treatment temperatures and times of naturally PFAS-contaminated soil from Stockholm Arlanda Airport, Sweden at fire training facility contaminated with PFAS-containing aqueous film-forming foam (AFFF)**

| **Treatment** | **Minutes** | **PFBA** | | **PFHxA** | | **PFOA** | | **PFNA** | | **PFUnDA** |  |  |
| --- | --- | --- | --- | --- | --- | --- | --- | --- | --- | --- | --- | --- |
| **temperature** | **of treatment** | **Medelvärde [mg/kg dw)]** | **SD** | **[mg/kg dw)]** | **SD** | **[mg/kg dw)]** | **SD** | **[mg/kg dw)]** | **SD** | **[mg/kg dw)]** | **SD** | |
| **150°C** | **15** | 0.0081 | 0.0022 | 0.0015 | 0.00029 | 0.0030 | 0.0011 | 0.0029 | 0.00017 | 0.0027 | 0.00013 | |
|  | **45** | 0.0029 | 0.00031 | 0.00063 | 0.00017 | 0.00071 | 0.00018 | 0.00023 | 0.000040 | 0.00097 | 0.00019 | |
|  | **75** | 0.0042 | 0.00029 | 0.00075 | 0.000060 | 0.0010 | 0.00011 | 0.00038 | 0.000050 | 0.0018 | 0.0010 | |
| **250°C** | **15** | 0.010 | 0.00074 | 0.0016 | 0.00023 | 0.0018 | 0.00022 | 0.00061 | 0.00019 | 0.0032 | 0.00049 | |
|  | **45** | 0.0055 | 0.0015 | 0.00085 | 0.00023 | 0.00082 | 0.00010 | 0.00026 | 0.000040 | 0.0011 | 0.00036 | |
|  | **75** | 0.0044 | 0.00054 | 0.00046 | 0.00011 | 0.00074 | 0.00035 | 0.00032 | 0.000080 | 0.0014 | 0.00061 | |
| **350°C** | **15** | 0.0062 | 0.0016 | 0.00066 | 0.00014 | 0.00070 | 0.00018 | 0.00026 | 0.00012 | 0.0014 | 0.00093 | |
|  | **45** | 0.0045 | 0.0012 | 0.00032 | 0.000070 | 0.00039 | 0.000010 | 0.00028 | 0.000060 | 0.0013 | 0.00045 | |
|  | **75** | 0.0037 | 0.00059 | 0.00073 | 0.00024 | 0.00080 | 0.00037 | 0.00078 | 0.00018 | 0.0019 | 0.00082 | |
| **450°C** | **15** | 0.0034 | 0.0012 | 0.00033 | 0.000020 | 0.00059 | 0.00030 | 0.00047 | 0.000090 | 0.0013 | 0.00026 | |
|  | **45** | 0.0045 | 0.00091 | 0.00042 | 0.00012 | 0.0015 | 0.00088 | 0.00040 | 0.00027 | 0.0013 | 0.00037 | |
|  | **75** | 0.0021 | 0.0017 | 0.00030 | 0.00016 | 0.00084 | 0.00070 | 0.00051 | 0.00030 | 0.00069 | 0.00035 | |
| **550°C** | **15** | 0.0012 | 0.0012 | 0.00019 | 0.000040 | 0.00044 | 0.00026 | 0.00037 | 0.00012 | 0.00080 | 0.000050 | |
|  | **45** | 0.00072 | 0.00020 | 0.00014 | 0.000060 | 0.00023 | 0.000040 | 0.00039 | 0.00013 | 0.0016 | 0.00054 | |
|  | **75** | 0.00039 | 0.00017 | 0.00013 | 0.000020 | 0.00022 | 0.000050 | <MDL | 0.000030 | 0.0011 | 0.000070 | |
|  |  | **PFHxS** | | **PFOS** | |  |  |  |  |  |  |  |
|  |  | **[mg/kg dw)]** | **SD** | **[mg/kg dw)]** | **SD** |  |  |  |  |  |  |  |
| **150°C** | **15** | 0.0016 | 0.00019 | 0.012 | 0.0024 |  |  |  |  |  |  |  |
|  | **45** | 0.00094 | 0.00035 | 0.0079 | 0.0015 |  |  |  |  |  |  |  |
|  | **75** | 0.0011 | 0.00012 | 0.010 | 0.00092 |  |  |  |  |  |  |  |
| **250°C** | **15** | 0.0016 | 0.000060 | 0.011 | 0.0020 |  |  |  |  |  |  |  |
|  | **45** | 0.0011 | 0.00011 | 0.014 | 0.0059 |  |  |  |  |  |  |  |
|  | **75** | 0.0012 | 0.00014 | 0.0094 | 0.0014 |  |  |  |  |  |  |  |
| **350°C** | **15** | 0.0011 | 0.000050 | 0.010 | 0.00053 |  |  |  |  |  |  |  |
|  | **45** | 0.0010 | 0.00013 | 0.011 | 0.00078 |  |  |  |  |  |  |  |
|  | **75** | 0.00090 | 0.000060 | 0.0072 | 0.00099 |  |  |  |  |  |  |  |
| **450°C** | **15** | 0.00049 | 0.00037 | 0.0044 | 0.0035 |  |  |  |  |  |  |  |
|  | **45** | <MDL |  | <MDL |  |  |  |  |  |  |  |  |
|  | **75** | <MDL |  | <MDL |  |  |  |  |  |  |  |  |
| **550°C** | **15** | <MDL |  | <MDL |  |  |  |  |  |  |  |  |
|  | **45** | <MDL |  | <MDL |  |  |  |  |  |  |  |  |
|  | **75** | <MDL |  | <MDL |  |  |  |  |  |  |  |  |

**Table S4. Averarage concentrations [mg PFAS/kg dry weight (dw)] and standard deviations (SD) of experimental triplicates after different treatment temperatures and times in sand soil from Högåsa Sweden fortified with 0.6 mg PFAS/kg dry dw**

| **Treatment** | **Minutes** | **PFBA** | | **PFHxA** | | **PFOA** | | **PFNA** | | **PFDA** | |
| --- | --- | --- | --- | --- | --- | --- | --- | --- | --- | --- | --- |
| **temperature** | **of treatment** | **[mg/Kg dw]** | **SD** | **[mg/Kg dw]** | **SD** | **[mg/Kg dw]** | **SD** | **[mg/Kg dw]** | **SD** | **[mg/Kg dw]** | **SD** |
| **150°C** | **15** | 0.21 | 0.051 | 0.39 | 0.029 | 0.68 | 0.033 | 0.54 | 0.024 | 0.23 | 0.035 |
|  | **45** | 0.24 | 0.024 | 0.35 | 0.053 | 0.51 | 0.0050 | 0.46 | 0.036 | 0.31 | 0.016 |
|  | **75** | 0.21 | 0.008 | 0.30 | 0.045 | 0.52 | 0.066 | 0.60 | 0.096 | 0.29 | 0.015 |
| **250°C** | **15** | 0.24 | 0.040 | 0.34 | 0.015 | 0.51 | 0.054 | 0.57 | 0.033 | 0.37 | 0.015 |
|  | **45** | 0.17 | 0.027 | 0.23 | 0.0050 | 0.39 | 0.065 | 0.31 | 0.0020 | 0.25 | 0.021 |
|  | **75** | 0.14 | 0.029 | 0.16 | 0.0040 | 0.30 | 0.038 | 0.15 | 0.0070 | 0.15 | 0.0020 |
| **350°C** | **15** | 0.090 | 0.003 | 0.12 | 0.031 | 0.16 | 0.049 | 0.095 | 0.016 | 0.079 | 0.0060 |
|  | **45** | 0.012 | 0.000 | 0.020 | 0.0010 | 0.013 | 0.0020 | 0.0080 | 0.0010 | 0.011 | 0.0020 |
|  | **75** | 0.0081 | 0.0013 | 0.0085 | 0.00090 | 0.0073 | 0.00060 | 0.0041 | 0.00060 | 0.0065 | 0.00080 |
| **450°C** | **15** | 0.0086 | 0.0031 | 0.0088 | 0.0036 | 0.0068 | 0.0018 | 0.0024 | 0.00040 | 0.0059 | 0.0015 |
|  | **45** | 0.00059 | 0.00016 | 0.00025 | 0.000036 | 0.00063 | 0.000058 | 0.00038 | 0.000013 | 0.0011 | 0.00029 |
|  | **75** | 0.00061 | 0.00029 | 0.00018 | 0.000045 | 0.00049 | 0.000010 | 0.00031 | 0.000057 | 0.00084 | 0.000096 |
| **550°C** | **15** | 0.00036 | 0.00015 | 0.00019 | 0.00010 | 0.00042 | 0.000063 | 0.00028 | 0.000027 | 0.00070 | 0.000021 |
|  | **45** | 0.00032 | 0.00013 | 0.00012 | 0.000033 | 0.00043 | 0.000046 | 0.00024 | 0.000038 | 0.00054 | 0.00012 |
|  | **75** | 0.00033 | 0.000025 | 0.00018 | 0.000025 | 0.00036 | 0.000043 | 0.00029 | 0.000095 | 0.00090 | 0.00023 |
|  |  | **PFUnDA** | | **PFHxS** | | **PFOS** | | **FOSA** | |  |  |
|  |  | **[mg/Kg dw]** | **SD** | **[mg/Kg dw]** | **SD** | **[mg/Kg dw]** | **SD** | **[mg/Kg dw]** | **SD** |  |  |
| **150°C** | **15** | 0.65 | 0.17 | 0.56 | 0.012 | 0.73 | 0.075 | 0.61 | 0.048 |  |  |
|  | **45** | 0.32 | 0.025 | 0.45 | 0.018 | 0.69 | 0.12 | 0.40 | 0.062 |  |  |
|  | **75** | 0.25 | 0.041 | 0.44 | 0.031 | 0.59 | 0.033 | 0.34 | 0.069 |  |  |
| **250°C** | **15** | 0.61 | 0.068 | 0.46 | 0.045 | 0.88 | 0.047 | 0.49 | 0.082 |  |  |
|  | **45** | 0.17 | 0.036 | 0.46 | 0.071 | 0.71 | 0.16 | 0.074 | 0.0060 |  |  |
|  | **75** | 0.12 | 0.032 | 0.46 | 0.040 | 0.70 | 0.023 | 0.034 | 0.0040 |  |  |
| **350°C** | **15** | 0.039 | 0.0020 | 0.39 | 0.070 | 0.63 | 0.059 | 0.026 | 0.0010 |  |  |
|  | **45** | 0.012 | 0.0010 | 0.28 | 0.023 | 0.36 | 0.034 | 0.0010 | 0.00001 |  |  |
|  | **75** | 0.0064 | 0.0016 | 0.21 | 0.0073 | 0.22 | 0.047 | <MDL |  |  |  |
| **450°C** | **15** | 0.0069 | 0.0026 | 0.23 | 0.058 | 0.23 | 0.094 | 0.00041 | 0.00028 |  |  |
|  | **45** | 0.00057 | 0.00045 | 0.00015 | 0.000041 | 0.00023 | 0.00028 | <MDL |  |  |  |
|  | **75** | 0.00061 | 0.00044 | <MDL |  | <MDL |  | <MDL |  |  |  |
| **550°C** | **15** | 0.00036 | 0.00026 | <MDL |  | <MDL |  | <MDL |  |  |  |
|  | **45** | 0.00042 | 0.00030 | 0.000083 | 0.00011 | <MDL |  | <MDL |  |  |  |
|  | **75** | 0.00069 | 0.00015 | 0.00015 | 0.000061 | <MDL |  | <MDL |  |  |  |
