# Supplementary material for: Thermal desorption as a high removal remediation technique for soils contaminated with per- and polyfluoroalkyl substances (PFASs)
Source: PLoS One. 2020 Jun 25;15(6):e0234476. doi: 10.1371/journal.pone.0234476 (PMC7316335; doi:10.1371/journal.pone.0234476)
Supplement: Attachment — Submitted filename: Respons to reviewers2.docx [file pone.0234476.s003.docx]

Response to reviewers

Reviewer #1: General
I would like to extend my thanks to the authors for their sincere effort to address the comments provided regarding the original version of the manuscript. I can see that the comments were well-received and that there was an attempt to address each one thoroughly. The result is an overall improvement; however, there are instances where I am not sure the intent of my comment was fully understood and/or where the resulting edits are somewhat confusing. To hopefully help address this I have tried to clarify some of these points in the line by line comments below.

**We sincerely thank the reviewers for the thorough comments to improve the manuscript**

Title/Abstract
• Line 19: Here and throughout the document, I recognize that the authors changed use of the word efficiency to rate in response to a prior comment, but I do not agree with use of the word rate. To me, removal rate implies a parameter with units that include time, and in this work it is being used to refer to the fraction of PFAS desorbed. I suggest changing to “fraction removed” or similar.

**Adressed**

• Line 20: The authors refer to “naturally contaminated soil.” Here and throughout the paper, I suggest that they modify this to field contaminated or similar. Naturally applies that the source of the impact is not anthropogenic in origin, and all PFAS are man-made.

**Adressed**

Introduction
• Lines 37-40: “The sentence beginning Thus, there is an urgent need to remediate….,” needs to be reworded. I am not certain if I understand the intent of the sentence, but I think it could be fixed by rewording it to say, “…PFAS-contaminated hotspot areas despite the fact that few soil guideline values…”

**Adressed**

• Line 42: I recommend changing “problem owners” here and throughout the manuscript to “responsible parties.”

**Adressed**

Results and Discussion
• Lines 141-142 state that removal rate generally increases with increasing temperature. In the PFAS fortified clay soil, the PFAS concentration at 150 has increased relative to the reference concentrations. Given that this soil was laboratory fortified, I assume that unknown precursors were not present in this sample that could lead to this increase. Were there background concentrations in the clay that were not originally accounted for?

**The background concentration was accounted for, and would have been shown in the untreated reference soil. The difference is within the measurement errors of the individual PFASs.**

• Lines 163-165: What about the potential effects of organic carbon?

**It is well known that PFAS partition to a higher extent to soil organic carbon, and the variable could have significant effect on the thermal desorption process. However, this effect was not isolated in this experiment and from the three different soils the effect could not be elucidated, and hence outside the scope of this study.**

• Line 170: I believe the authors mean to say “With the exception of PFBA…”

**Adressed**

• Lines 172-173: “This indicates that the main removal mechanism observed in this study is thermal desorption and not degradation.“ The lack of increase in shorter chain homologues is not sufficient evidence to support desorption over degradation. For example, what if the process generated short-chain PFAS smaller than those monitored in this project?

**From the previous studies by our research group** ^1^**, and other groups** ^2^**, degradation occurs by stepwise defluorination and increase of some of shorter chained PFASs included in the study is normally observed (i.e. PFBA and PFPeA) degradation products from longer chained PFASs. We can not say for sure that no ultra-short chained PFASs have been formed, but there was no increase in i.e. PFBA or PFPeA or other PFASs and hence no indications of PFAS degradation. Hence, we opt to keep the sentence as it is.**

• Lines 177-178 state that the optimal treatment temperature is 350-450oC; however line 182 says that PFAS-impacted soils can be treated using “thermal desorption at temperatures above 450oC. This is contradictory.

**Adressed**

• Lines181-182: In the previous version of the manuscript, I asked that the authors define their criteria for efficient treatment. This had the unintended consequence of leading the authors to change all uses of “efficient” in the manuscript. As mentioned above, I do not think the use of the word rate herein is the best way to express what the authors actually evaluated which is the fraction removed and not, for example, first order rate coefficients. In this specific sentence the authors now say soils can be treated with “high removal,” which doesn’t address the issue. What are their criteria defining high? I recommend the authors stick to the facts of their data. For example, they might say that this treatment technique is capable of removing up to x% of PFAS in soils.

**The authors agree with the reviewer that “removal rates” and “removal efficiency” can be misleading concepts, and have subsequently replaced the term with either “removal” or “fraction removed”.**

**However, in comparison to multiple other studies and the long experience of the authors of working with PFAS remediation in both water and soil matrices, the authors consider removal rates of >99% to be relatively high removal compared to other techniques. This is an important finding of the study, and hence the authors keep the “high removal” expression, while also throughout the manuscript the absolute removal is clearly expressed in %.**

**1.	Franke, V., Schäfers, M. D., Lindberg, J. J. & Ahrens, L. Removal of per- And polyfluoroalkyl substances (PFASs) from tap water using heterogeneously catalyzed ozonation. *Environ. Sci. Water Res. Technol.* 5, 1887–1896 (2019).**

**2.	Liu, Y. *et al.* Efficient Mineralization of Perfluorooctanoate by Electro-Fenton with H2O2 Electro-generated on Hierarchically Porous Carbon. *Environ. Sci. Technol.* 49, 13528–13533 (2015).**
